# Prevalence of *Neospora caninum* antibodies in fattening pigs and sows from intensive farms in northern Italy

**DOI:** 10.1007/s00436-022-07457-z

**Published:** 2022-02-04

**Authors:** Luca Villa, Alessia Libera Gazzonis, Carolina Allievi, Sergio Aurelio Zanzani, Michele Mortarino, Maria Teresa Manfredi

**Affiliations:** Department of Veterinary Medicine, Università Degli Studi Di Milano, Via dell’Università 6, 26900 Lodi, Italy

**Keywords:** Neosporosis, Swine, Intensive farms, IFAT, Biosecurity, Reproductive problems

## Abstract

*Neospora caninum* (Apicomplexa, Sarcocystidae) is a major cause of reproductive failure in cattle. In pigs, only a few studies investigated the effects of this parasite on reproductive efficiency. Considering the relevance of swine farms in northern Italian regions, an epidemiological survey was designed to investigate the spread of *N. caninum* infection. Three hundred seventy fattening pigs and sows from 23 intensive farms in Lombardy were sampled. Sera were analyzed by a commercial immunofluorescence antibody test. Statistical analysis through univariate and multivariate generalized linear models was conducted to detect farm management practices enhancing the risk of infection. At the farm level, 52.1% (12/23) of the selected farms, 72.7% housing sows and 40% fattening pigs, scored positive. At the individual level, 25 animals (25/370, *P* = 6.7%) were positive to *N. caninum* antibodies: one fattening pig and two sows showed an antibody titer of 1:100, and in two sows, an antibody titer of 1:400 and 1:6400 was evidenced. A higher seroprevalence was detected in sows (17/151, *P* = 11.2%) if compared to fattening pigs (8/219, *P* = 3.6%) (OR = 1.19, *P* value = 0.000 in sows). Moreover, a higher seroprevalence was recorded in farms with low and moderate sanitary score (*P* = 100% and *P* = 64.2%, respectively) if compared to farms with high sanitary score (*P* = 22.2%) (OR = 1.24, *P* value = 0.007 in score = 1 and OR = 1.10, *P* value = 0.050 in score = 2). This study provides the first data on the circulation of *N. caninum* in intensive swine farms in Italy, demonstrating the spread of the parasite in fattening pigs and sows in Lombardy region.

## Introduction

*Neospora caninum*, an obligate intracellular protozoan, is the causative agent of neosporosis, a severe clinical disease of cattle and dogs worldwide (Dubey 2003). Serological evidence in domestic and wild animals indicates that many species were exposed to this parasite (Almería and López-Gatius 2013). Domestic dogs and wild canids are the definitive hosts; various species were reported as intermediate hosts of the parasite, including ruminants, equids, and swine (Dubey, 2003). *N. caninum* is a major cause of abortion, the main clinical manifestation of bovine neosporosis, which causes huge economic losses to the dairy and beef industries worldwide (Thilsted and Dubey 1989; Goodswen et al. 2013).

In pigs, few studies investigated the effects of *N. caninum* infection on reproductive efficiency in sows. A major concern in swine farming are reproductive disorders; indeed, the drop in piglets/sow/year causes economic losses. A variety of pathogens are recognized as responsible for reproductive disorders in pigs: however, the pathogenesis of neosporosis and its consequences in the swine species remain unclear (Snak et al. 2019, 2021). A study evidenced an influence of *N. caninum* seropositivity on reproductive parameters in sows, i.e., age at first farrowing, the annual number of deliveries, and stillbirth incidence (Kamga-Waladjo et al. 2009). Recently, Snak et al. (2019) demonstrated that in experimentally infected pigs, the parasite could be trans-placentally transmitted in all phases of gestation, regardless of the time of infection, causing reproductive disorders and abortion with mummified fetuses, especially in the first and second gestational thirds. Besides, due to reactivation of the infection, the endogenous vertical transmission was evidenced in the sows inoculated in the final third of gestation. Moreover, *N. caninum* can cause clinical signs in infected female pigs, including hypothermia and leukocytosis in the acute phase of infection; the infection can also acutely reappear in chronically infected swine during pregnancy (Snak et al. 2021).

The occurrence of *N. caninum* natural infection in pigs was reported in some countries throughout the world with varying antibody prevalence depending on the region, the production system, and the diagnostic test employed (Wyss et al. 2000; Damriyasa et al. 2004; Helmick et al. 2002; Azevedo et al. 2010; Bártová and Sedlák 2011; Feitosa et al. 2014; Minetto et al. 2019; Silva et al. 2019; Gui et al. 2020).

Regarding Italy, *N. caninum* infection was reported in various species, i.e., in cattle (Otranto et al. 2003; Rinaldi et al. 2005; Villa et al. 2021) and other domestic species, including dogs, cats, equids, and small ruminants (Ferroglio et al. 2005, 2007a; Villa et al. 2018; Gazzonis et al. 2020), but also in wild mammals and birds (Ferroglio and Rossi 2001; Ferroglio et al. 2007b; Zanet et al. 2013; Gazzonis et al. 2021).

To date, no data on *N. caninum* infection in swine is available for Italy. Considering the relevance of swine farms under the intensive production system in northern Italian regions, this study aimed to investigate the spread of *N. caninum* infection in intensively reared fattening pigs and sows in Lombardy region. Besides, another aim was to evaluate the association between the seroprevalence of *N. caninum* and animal husbandry practices and farm biosecurity procedures to identify potential critical points of the farm system favorable to the parasite circulation.

## Materials and methods

### Sample collection

The sampling of fattening pigs and sows was performed as previously described for an epidemiological survey on *Toxoplasma gondii* infection in pigs from intensive production system (Gazzonis et al. 2018a).

The survey was carried out in Lombardy, one of the most suitable regions for intensive pig farming in northern Italy. Overall, 219 fattening pigs and 151 sows from 15 and 11 conventional farms, respectively, were sampled at five slaughterhouses; in three farms (Farm.02, Farm.13, and Farm.17), the collection of both sow and fattening pig samples was feasible. For each farm, an average of 16 individuals was sampled (min–max, 3–34). During the slaughtering operations, for each animal, a blood sample was collected from a jugular vein into tubes without anticoagulants. Blood samples were transported to the laboratory within a few hours; blood was centrifuged (15 min, 2120 × g), and serum was transferred into Eppendorf tubes and stored at − 20 °C until serological analysis.

At sampling time, data on farm management were collected, and a “biosecurity score” (ranging from 1 = poor, 2 = moderate, to 3 = optimal) was determined for each farm based on parameters regarding the sanitary procedures applied, as previously described (Gazzonis et al. 2018a).

### Serological analysis

**Fig. 1:**
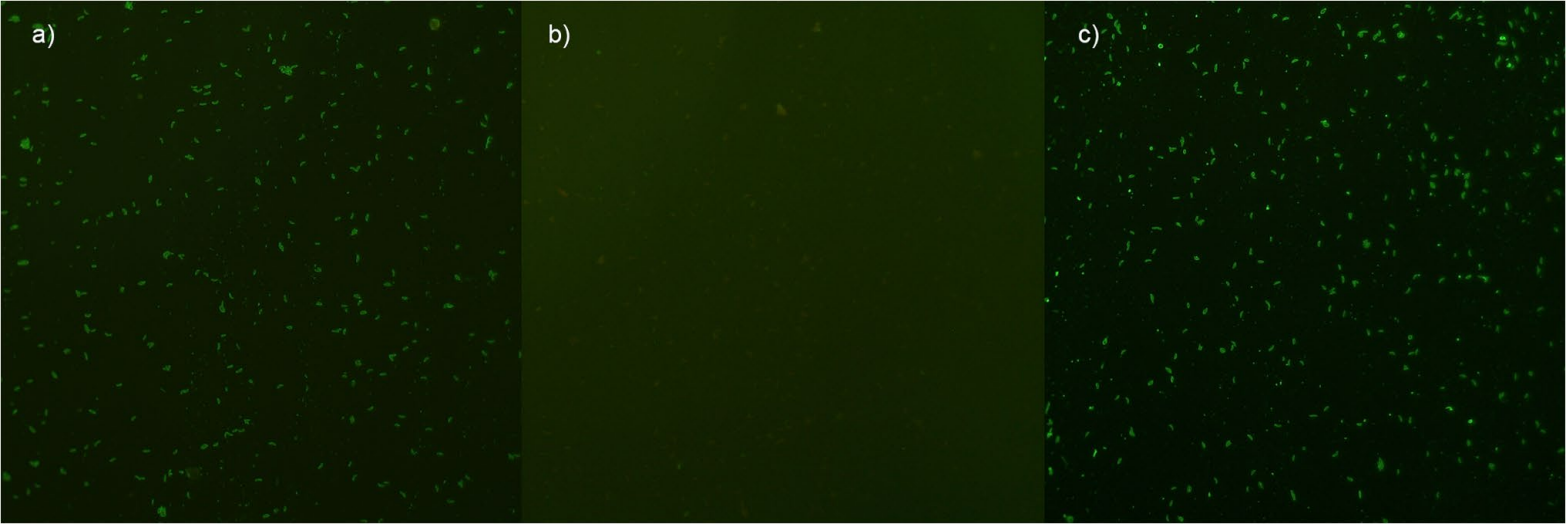
Immunofluorescence detection images (100 ×) of **a** positive control, **b** negative control, and **c** one positive sow with an antibody titer of 1:6400 at 1:160 dilution

Sera samples were analyzed for anti-*N. caninum* antibodies by immunofluorescence antibody test, using slides coated with *Neospora caninum* antigens provided in a commercial kit (MegaScreen® FLUO NEOSPORA caninum, Megacor, Austria), following the manufacturer’s instruction, with slight modifications. An initial screening dilution of 1:50 of serum in PBS was used according to Snak et al. (2019); then, seropositive samples were twofold serially diluted to determine the end-point antibody titer. Briefly, 20 μl of serum dilutions were pipetted on separate antigen wells; 20 μl of the positive and negative controls supplied in the kit were also included in each assay. The slides were incubated for 30 min at 37 °C in a humid chamber. After a washing step with PBS, one drop (20 μl) of FITC anti-pig IgG as conjugate (MegaFLUO® FITC anti-pig IgG Conjugate, Megacor, Austria) was placed onto each well. A subsequent incubation step for 30 min at 37 °C in the dark followed by another washing step was performed. Finally, some drops of mounting medium were added to the coverslips, which were then placed on the slides. The slides were evaluated using a fluorescence microscope (Axioscope 2, Zeiss), comparing each well to the fluorescence of the positive and negative controls, considered as a reference pattern. Only a bright, sharp, and clear, yellow-green fluorescence on the membrane extending to the whole body of *N. caninum* tachyzoites was considered a positive reaction (Fig. 1).

### Statistical analysis

Seroprevalence of *N. caninum* infection was calculated at the individual and farm levels according to the considered categories. A farm was considered positive if at least one sampled animal scored positive. Serological data were analyzed to determine which variables could be predictors of *N. caninum* infection; the farm was considered as the statistical unit. Overall, 26 observations were included in the analysis. The only three farms where both sows and fattening pigs were sampled, were defined as independent units, since management could considerably vary within the same farm according to the considered productive category.

Separate generalized linear models (GLMs) with negative binomial distribution were performed to verify the influence of farm management on *N. caninum* infection. The intra-herd seroprevalence was considered as the dependent variable; the variables listed in Table 1 were entered as independent variables in the univariate model. All variables showing a *P* value < 0.1 were entered in multivariate models developed through a backward selection procedure (significance level to remove variables from the model = 0.05), based on Akaike’s information criterion (AIC) values. Besides, in the final models, the estimated prevalence values of the biosecurity score levels were compared through pairwise comparisons. Statistical analysis was performed using IBM SPSS Statistics for Windows version 25.0 software (IBM Corp., Armonk, NY, USA).

**Table 1:** Baseline characteristics related to farm management of pig farms and *Neospora caninum* seroprevalence values for each considered category

Variable	Category	Positive/examined farms	P% (95% CI)
Productive category	Sows	8/11	72.7 (43.44–90.25)
	Fattening pigs	6/15	40.0 (19.82–64.25)
Number of pigs in the farm	≤ 1000	2/8	25.0 (7.15–59.07)
	1000–2500	4/10	40.0 (16.82–68.73)
	≥ 2500	4/8	50.0 (21.52–78.48)
Floor type	Fully slatted	1/2	50.0 (9.45–90.55)
	Partly slatted	1/9	11.1 (1.99–43.50)
	Straw bedding	5/15	33.3 (15.18–58.29)
Animal density (m^2^)	1	4/8	50.0 (21.52–78.48)
	1–2	3/11	27.2 (9.75–56.56)
	≥ 2	3/7	42.8 (15.82–74.95)
Possibility to access outdoors	Yes	5/15	33.3 (15.18–58.29)
	No	5/11	45.5 (21.27–71.99)
Presence of rodents	Yes	14/26	53.8 (35.46–71.24)
	No	0/0	0 ( −)
Application of pest control	Only internal	3/7	42.8 (15.82–74.95)
	Internal and external	7/19	36.8 (19.15–58.96)
Sanitary score	1	3/3	100 (43.85–100)
	2	9/14	64.2 (38.76–83.66)
	3	2/9	22.2 (6.32–54.74)

## Results

### Serological analysis

Overall, 370 swine from 23 farms, including 219 fattening pigs from 15 farms and 151 sows from 11 farms, were analyzed for anti-*N. caninum* antibodies. At the farm level, 52.1% (12/23) of the selected farms hosted at least one positive animal; in particular, 72.7% of the farms housing sows and 40% of those housing fattening pigs included in the study scored positive. Serological results for each examined farm are reported in Table 2. At the individual level, 25 animals (25/370, *P* = 6.7%, 95% CI 4.62–9.78) were positive to *N. caninum* at the initial screening dilution. A higher seroprevalence was detected in sows (17/151, *P* = 11.2%, 95% CI 7.15–17.29) compared to fattening pigs (8/219, *P* = 3.6%, 95% CI 1.86–7.04). The intra-herd seroprevalence in *N. caninum* infected farms varied between 13.3 and 60% considering sows and between 3.3 and 13.3% in fattening pigs (*P* = 23% and *P* = 3%, respectively). Moreover, a higher seroprevalence was recorded in farm with a low sanitary score (*P* = 100% and *P* = 64.2% in farms with score = 1 and score = 2, respectively) if compared to farms with higher sanitary score (*P* = 22.2% in farms with score = 3). Among seropositive animals, one fattening pig and two sows showed an antibody titer of 1:100; besides, an antibody titer of 1:400 and 1:6400 was evidenced in two sows, respectively. The serological results related to farm characteristics are reported in Table 1.

**Table 2:** *Neospora caninum* infection in pigs in northern Italy: serological results including antibody titers reported for each examined farm

Farm	No. of positive/examined animals (antibody titer)		
	Fattening pigs	Sows	Total
1	-	3/7 (1:50, 1:50, 1:100)	3/7
2	1/30 (1:50)	2/4 (1:50, 1:6400)	3/34
3	1/12 (1:50)	-	1/12
4	0/10	-	0/10
5	0/18	-	0/18
6	0/18	-	0/18
7	-	0/30	0/30
8	-	3/5 (1:50, 1:100, 1:400)	3/5
9	0/5	-	0/5
10	0/10	-	0/10
11	2/15 (1:50, 1:100)	-	2/15
12	-	0/4	0/4
13	0/10	2/30 (1:50)	2/40
14	-	1/4 (1:50)	1/4
15	-	4/30 (1:50)	4/30
16	-	0/30	0/30
17	1/25 (1:50)	1/4 (1:50)	2/29
18	0/10	-	0/10
19	2/18 (1:50)	-	2/18
20	0/10	-	0/10
21	1/18 (1:50)	-	1/18
22	0/10	-	0/10
23	-	1/3 (1:50)	1/3
Total	8/219	17/151	25/370
Prevalence (%)	3.65	11.25	6.75

### Statistical analysis

By univariate analysis, only the variables “productive category” and “biosecurity score” were significantly associated with *N. caninum* infection and were entered in the final multivariate model (Table 3). Indeed, sows were at a higher risk of infection than fattening pigs (OR = 1.19, *P* value = 0.000). Moreover, the biosecurity score was a predictor of infection, increasing the risk of infection while decreasing the score (OR = 1.24, *P* value = 0.007 and OR = 1.10, *P* value = 0.050 in score = 1 and score = 2, respectively). Besides, the pairwise comparison revealed that seroprevalence values of farms with biosecurity score = 1 and score = 2 were statistically different from those of score = 3 (*P* value < 0.05).

**Table 3:** Results of the multivariate analysis of the risk factors related to *Neospora caninum* seroprevalence in pigs in Lombardy

Response variable	Category	P%	β ^a^	Standard error of coefficients	Wald Chi-square	Odds ratio (95% confidence interval)	*P* value	Akaike information criterion
Productive category	Sows	72.7	0.18	0.05	13.43	1.19 (1.09–1.31)	0.000	15.63
	Fattening pigs	40.0	0			1		
Sanitary score	1	100 _a_	0.22	0.08	7.17	1.24 (1.06–1.45)	0.007	
	2	64 _a_	0.10	0.05	3.83	1.10 (1.00–1.22)	0.050	
	3	22	0			1		

Seroprevalence values according to each variable followed by the lowercase letter “a” are statistically different from other values without the lowercase letter “a” at *P* value < 0.05 (generalized linear models’ analysis, pairwise comparison), while they are not statistically different from each other (*P* value > 0.05, GLM, pairwise comparison)

^a^Coefficient

## Discussion

This study provides the first data on the circulation of *N. caninum* in intensive swine farms in Italy. A seroprevalence of 6.7% was evidenced at the individual level; at the farm level, 52.1% of the farms were positive. In the present survey, a cut-off of 1:50 for IFAT was applied, according to previous studies (Paré et al. 1995; Snak et al. 2019). Some animals presented higher antibody titer: in particular, one fattening pig and two sows showed an antibody titer of 1:100 and two sows of 1:400 and 1:6400, respectively.

It was evidenced that breeder animals were at higher risk of acquiring the parasite infection: sows were more frequently found seropositive (*P* = 11.2%) if compared to fattening pigs (*P* = 3.6%), also considering both the farm level (*P* = 72.7% and *P* = 40.0%, respectively) and the mean intra-herd seroprevalence (*P* = 23% and *P* = 3%, respectively). Indeed, sows are slaughtered at an older age (3–5 years or more) than fattening pigs (approximately 9 months), increasing their risk of acquiring the infection through environmental, horizontal transmission. The same finding was previously revealed in pigs also in other studies (Wyss et al. 2000; Silva et al. 2019; Gui et al. 2020).

In addition to individual characteristics, some features concerning farm management, including sanitary procedures, were associated with *N. caninum* infection. Risk factor analysis highlighted that the farms with a low biosecurity level presented a higher risk of being infected. According to previous studies which demonstrated the association between sanitary procedures and seropositivity for *T. gondii* (Gazzonis et al. 2018a), farms with a poor or moderate sanitary score recorded a seroprevalence of *N. caninum* of 100% and 64.2%, whereas those with a good biosecurity level evidenced a seropositivity of 22.2%. The adoption of adequate hygiene, prevention measures, and biosecurity protocols, including the application of a health management program, vaccination protocols, standard protocols for quarantine for imported animals, protocols for visitors/transporters, sanitary protocols for operators, and application of an all-in/all-out system, proved once again to be fundamental to prevent the risk of infections in the farm, including parasitic infections.

This is the first study investigating *N. caninum* infection in swine species in Italy. However, considering the Italian epidemiological scenario, previous studies reported the circulation of the parasite in other species. Indeed, in cattle, serological studies evidenced a herd prevalence of 44.1% and 77.8% and individual seropositivity values of 11% and 30.8%, respectively (Otranto et al. 2003; Rinaldi et al. 2005). A recent survey reported a molecular prevalence of *N. caninum* of 27.8% in aborted bovine fetuses in cattle farms in Lombardy region, confirming that the parasite circulates in the study area (Villa et al. 2021). Concerning small ruminants, Gazzonis et al. (2020) reported herd and individual prevalence values of 89.4% and 19.3% in sheep and 32.1% and 5.7% in goats in Lombardy, respectively. Moreover, other domestic species in northern Italian regions were also exposed to the parasite with variable seroprevalence values, i.e., 30.2% in dogs (Ferroglio et al. 2007a), 31.9% in cats (Ferroglio et al. 2005), and 0.4% in horses (Villa et al. 2018). Among wild mammals, Ferroglio and Rossi (2001) confirmed serological exposure to *N. caninum* in wild ruminants (5.9% in chamois, 2.3% in roe deer, and 1.9% in red deer), whereas parasitic DNA was detected in 10.3% of rodents (Ferroglio et al. 2007b) and 2.8% of eastern cottontail rabbits (Zanet et al. 2013); besides, Gazzonis et al. (2021) reported a molecular prevalence of 3.6% in wild birds in northern Italy. All these data confirm the circulation of *N. caninum* in both domestic and wild animals in Italy.

To compare seroprevalence data, differences in study populations, diagnostic techniques, and protocols should always be considered. The values recorded in other European studies resulted similar but lower than those of our study: 1–3% in Switzerland (Wyss et al. 2000), 3% in the Czech Republic (Bártová and Sedlák 2011), 0.04% in Germany (Damriyasa et al. 2004), and 0% in England (Helmick et al. 2002). Outside Europe, some studies were conducted in Brazil, where the seroprevalence varied between 3.1–3.2% (Azevedo et al. 2010; Feitosa et al. 2014), 13.5% (Minetto et al. 2019), and 18.9% (Silva et al. 2019). Moreover, a study from China recorded a prevalence of 1.9%, and parasitic DNA was detected in the brain tissues of three pigs (Gui et al. 2020).

To date, only a few studies investigated the effects of *N. caninum* infection in sows. A survey on wandering sows in Senegal evidenced an influence of *N. caninum* seropositivity on reproductive parameters, i.e., age at first farrowing, the annual number of deliveries, and stillbirth incidence (Kamga-Waladjo et al. 2009).

Furthermore, serological studies on the spread of *N. caninum* were also conducted in European wild boars’ populations (Almería et al. 2007; Bártová et al. 2006; Reiterová et al. 2016). Considering that wild boar populations are nowadays in expansion in terms both of the number of animals and habitat range, the increased frequency of contacts among wild boars, livestock, and humans could influence the transmission of zoonotic and animal-specific pathogens, as previously demonstrated in this area for other similar protozoa infections (Gazzonis et al. 2018b, 2019a). In the same study area, a recent molecular survey reporting the presence of *N. caninum* in brain tissue of wild birds of prey not only suggests the involvement of avian species in the parasite life cycle but also the environmental circulation of *Neospora*, indicating a possible role of these wild populations in the epidemiology of the parasite infection at the interface of the domestic and sylvatic life cycle (Gazzonis et al. 2021).

Indeed, in analogy to other intermediate hosts, domestic pigs probably become infected with *N. caninum* by ingesting food or drinking water contaminated by sporulated oocysts shed from dogs or tissues containing cysts of other intermediate hosts (e.g., micromammals). Besides, the parasite may be transmitted trans-placentally (congenital vertical transmission) from an infected dam to the fetuses during pregnancy, as experimentally demonstrated (Jensen et al., 1998; Snak et al. 2019).

Experimental studies evidenced that all pigs seroconverted after inoculation with Nc1 strain (Dubey et al. 1996; Jensen et al. 1998; Snak et al. 2019, 2021). Moreover, Jensen et al. (1998) also detected the parasite in two fetuses providing the first indication of transplacental transmission of *N. caninum* in pigs. A recent study (Snak et al. 2019) confirmed that in pigs, *N. caninum* could be transmitted via the placenta, causing reproductive disorders, in particular mummified fetuses, especially in the first and second gestational thirds. Furthermore, in the same study, parasitic DNA was detected in milk and amniotic fluid, suggesting a role of these matrices in the protozoan transmission, as also evidenced for both *T. gondii* and *N. caninum* in other species, including ruminants and equids (Björkman et al. 1997; González-Warleta et al. 2011; Mancianti et al. 2014; Gazzonis et al. 2018c, 2019b). It was also demonstrated that *N. caninum* can cause clinical signs in adults; indeed, experimentally infected sows showed hyperthermia followed by hypothermia and leukocytosis in the acute phase (Snak et al. 2021).

## Conclusions

*N. caninum* circulates widely in the pig farms of northern Italy, where natural parasite infection occurs both in fattening pigs and in sows. Considering the importance of pig farming in the study area, linked to valuable production, the impact of *N. caninum* infection in intensive pig farming should be further investigated, particularly in breeding sows, due to its possible involvement in reproductive problems. Indeed, the correct diagnosis of the cause of abortion is important for adopting appropriate control and prevention measures. Among the further perspectives, the molecular detection and multilocus microsatellite genotyping of *N. caninum* would indicate the spatial distribution and mutual connections between the parasite’s isolates from different species.

## Data Availability

The data that support the findings of this study are available from the corresponding author upon reasonable request.
